# A Cross-sectional Assessment of Health-related Quality of Life among Patients with Chronic Obstructive Pulmonary Disease

**Published:** 2017-08

**Authors:** Miguel Ángel GARCIA-GORDILLO, Daniel COLLADO-MATEO, Pedro Rufino OLIVARES, José Carmelo ADSUAR, Eugenio MERELLANO-NAVARRO

**Affiliations:** 1. Dept. of Economics, Faculty of Economics and Business Sciences, University of Extremadura, Badajoz, Spain; 2. Dept. of Applied Economics, Faculty of Economics and Business, University of Murcia, Murcia, Spain; 3. Faculty of Sport Sciences, University of Extremadura, Cáceres, Spain; 4. Instituto de Actividad Fisica y Salud, Universidad Autonoma de Chile, Talca, Chile; 5. Higher Institute of Physical Education, University of the Republic, Montevideo, Uruguay

**Keywords:** EQ-5D, Quality of life, COPD, Pulmonary disease, Normative values

## Abstract

**Background:**

Chronic obstructive pulmonary disease (COPD) is a major cause of mortality characterized by progressive airflow obstruction and inflammation in the airways, which has an impact on health-related quality of life. The EQ-5D-5L is one of the most used preference-based, health-related quality of life questionnaire. The objective of this study was to provide normative values of EQ-5D-5L for Spanish people suffering from COPD.

**Methods:**

Data were extracted from the Spanish National Health Survey (2011/2012). Overall, 1130 people with COPD participated in this survey. The utility index of EQ-5D-5L and the Visual Analog Scale (VAS) score were defined by gender, region, and age.

**Results:**

Mean (SD) EQ-5D-5L utility index and VAS score for Spanish people with COPD were 0.742 (0.309) and 60.466 (21.934) respectively. In general, men reported better health status than women. Ceiling effect of the whole sample was 30.35%.

**Conclusion:**

The current study provides normative values of EQ-5D-5L for Spanish people affected by COPD. Ceiling effect was high and better results were observed in men compared with women.

## Introduction

Chronic obstructive pulmonary disease (COPD) is a major cause of morbidity and mortality worldwide, characterized by progressive airflow obstruction and inflammation in the airways (1). According to the World Health Organization, it is not one single disease but an umbrella term, which includes chronic lung diseases that affect the airflow. In this regard, chronic bronchitis and emphysema are now included within the COPD diagnosis.

The estimated prevalence of COPD in Spanish adults aged 40–80 years is 10.2% and is higher in men (15.6%) than in women (5.6%). This prevalence is increased with age and with cigarette smoking (2). COPD is associated with reduced health-related quality of life (HRQoL) but the reduction is stronger on the physical than on the mental component of HRQoL. The impact of severe COPD on HRQoL is higher than the reported impact of other diseases such as diabetes or self-reported cardiovascular diseases (3). Comorbidities in COPD are also associated with worse HRQoL and excess in costs, especially, cardiovascular diseases, depression, anxiety and diabetes (4, 5). COPD imposes a substantial burden. According to the study in Spain, the total cost per patient per year was €1922.60 (6). Of that amount, hospitalization costs were the highest with €788.72; followed by cost of drugs, €492.87; and emergencies, €134.32.

The EQ-5D-5L (7) is one of the most used tools to evaluate HRQoL. It was developed from the previous version of EQ-5D, which only included 3 levels of problem (8). The questionnaire also includes a Visual Analogue Scale (VAS), on which the best imaginable health state is marked 100 and the worst is marked 0.

There are few studies using EQ-5D-5L in patients with COPD. A multi-country (Denmark, England, Italy, Netherlands, Poland, and Scotland) study compared the properties of EQ-5D-3L and EQ-5D-5L across 8 patient groups, including respiratory disease (COPD or asthma) (9). In that study, absolute discriminatory power had remarkably improved with EQ-5D-5L.

Normative values for a specific region and condition are often useful in the interpretation of results by other researchers, taking into account deviations according to age, gender or other variables. In this regard, there is a lack of normative values for Spanish people suffering from COPD. Therefore, the main objective of the current study is to provide the normative values of EQ-5D-5L from a representative Spanish sample with COPD.

## Methods

The current cross-sectional study used data from the Spanish National Health Survey. This survey is periodically conducted by the Spanish Ministry of Health, Social Services, and Equality. Acquisition of data was performed between Jul 2011 and Jun 2012. The method utilized to collect data was computer-assisted personal interviews (CAPI). The mentioned survey included the EQ-5D-5L in the health status block for the first time since it is performed.

The sample of the Spanish National Health Survey is representative for the Spanish population and for the 17 autonomous regions and the 2 autonomous cities. Totally, 21007 participants completed the survey. Of these, 1130 (15–102 yr) were diagnosed with EPOC (including emphysema and chronic bronchitis).

### Statistical analysis

The current study presents descriptive statistics (mean, SD, median, interquartile range –IQR- and ceiling effect) of EQ-5D-5L utility index and VAS. The whole sample was stratified by gender, age groups, and 19 regions. Potential influence of marital status, smoking status, net monthly income of household, and educational level were also considered.

The 5-digit EQ-5D-5L health status and the VAS were obtained from the Spanish National Health Survey. The health status 11111 would be the perfect health state, whereas 55555 would mean the worst imaginable health state. EQ-5D-5L utility was calculated from the 5-digit health status score by using the algorithm available at the website of the EuroQol Group (https://euroqol.org/). In Spanish population, this algorithm to calculate EQ-5D-5L utility is the result of a “crosswalk” from the version with 3 levels. The EQ-5D-5L utility index for Spanish population can range from −0.654 (worst imaginable health status) to 1 (perfect health status).

Therefore, ceiling effect can be calculated as the frequency of the health status 11111, whereas the floor effect would be the opposite, i.e. the frequency of the health status 55555. Given that the floor effect is not reported in the EQ-5D-5L, the current study only evaluates the frequency (total number and percentage) of the perfect health state in order to calculate the ceiling effect.

Mann-Whitney U and Kruskal-Wallis H non-parametric tests were used in the analysis of the subgroups. A p-value 0.05 was set for all the tests in order to indicate statistical significance. The answers “do not know” and “no answer” were considered as missing data.

## Results

The mean and SD of EQ-5D-5L utility and the VAS score can be seen in Table 1.

**Table 1:** Study sample characteristics EQ-5D-5L population norms

	**n (%)**	**EQ-5D-5L Index**		**EQ-5D-VAS**		**Ceiling effect**	***P*-value**
		**Mean (SD)**	**Median (IQR)**	**Mean (SD)**	**Median (IQR)**	**n (%)**	
**Overall**	1,130 (−)	0.74 (0.31)	0.85 (0.38)	60.47 (21.93)	61 (29)	343 (30.35)	
**Gender**							**<0.001** ^a^
Male	550 (48.67)	0.8 (0.28)	0.91 (0.3)	61.87 (21.66)	65 (29)	206 (37.45)	
Female	580 (51.33)	0.69 (0.33)	0.8 (0.39)	59.16 (22.13)	60 (30)	137 (23.62)	
**Age group**							**<0.001** ^b^
15–39	129 (11.42)	0.94 (0.10)	1 (0.09)	76.64 (19.22)	81 (21)	87 (67.44)	
40–65	397 (35.13)	0.80 (0.25)	0.90 (0.26)	62.8 (20.81)	64 (29)	135 (34.01)	
66–102	604 (53.45)	0.66 (0.34)	0.74 (0.40)	55.3 (21.23)	55 (29.5)	121 (20.03)	
**Region**							**0.09** ^b^
Andalusia	123 (10.88)	0.68 (0.35)	0.83 (0.39)	55.94 (21.75)	55 (32)	25 (20.33)	
Aragon	42 (3.72)	0.73 (0.32)	0.84 (0.37)	59.26 (17.88)	55 (20.25)	14 (33.33)	
Principality of Asturias	64 (5.66)	0.7 (0.33)	0.78 (0.41)	59.16 (19.86)	60 (26.5)	19 (29.69)	
Balearic Island	31 (2.74)	0.78 (0.25)	0.89 (0.4)	65.35 (25.81)	74 (41)	12 (38.71)	
Canarias	74 (6.55)	0.69 (0.3)	0.78 (0.39)	59.3 (21.13)	60 (26)	14 (18.92)	
Cantabria	29 (2.57)	0.63 (0.43)	0.89 (0.7)	49.9 (19.04)	50 (23)	9 (31.03)	
Castile and Leon	73 (6.46)	0.82 (0.18)	0.84 (0.3)	58.9 (21.12)	60 (37)	22 (30.14)	
Castile-La Mancha	69 (6.11)	0.66 (0.4)	0.85 (0.63)	57.41 (24.78)	60 (41)	24 (34.78)	
Catalonia	149 (13.19)	0.77 (0.26)	0.86 (0.4)	64.13 (21.89)	69 (30)	42 (28.19)	
Community of Valencia	83 (7.35)	0.73 (0.33)	0.83 (0.33)	60.92 (20.04)	61 (26)	28 (33.73)	
Extremadura	56 (4.96)	0.82 (0.2)	0.88 (0.29)	55.4 (23.59)	59 (31)	18 (32.14)	
Galicia	72 (6.37)	0.72 (0.33)	0.86 (0.43)	61.1 7(20.1)	64 (24)	20 (27.78)	
Community of Madrid	78 (6.9)	0.76 (0.29)	0.89 (0.32)	64.5 (23.71)	69 (35)	24 (30.77)	
Murcia Region	51 (4.51)	0.81 (0.22)	0.88 (0.34)	62.71 (22.73)	64 (31)	19 (37.25)	
Community of Navarre	47 (4.16)	0.81 (0.23)	0.86 (0.27)	61.51 (18.01)	66 (22)	12 (25.53)	
Basque Country	56 (4.96)	0.72 (0.39)	0.89 (0.36)	65.14 (22.66)	70 (32.25)	24 (42.86)	
La Rioja	19 (1.68)	0.88 (0.24)	1 (0.14)	77.16 (18.21)	84 (20)	12 (63.16)	
Ceuta	9 (0.8)	0.85 (0.17)	0.91 (0.31)	60.11 (29.27)	71 (43.5)	3 (33.33)	
Melilla	5 (0.44)	0.82 (0.22)	0.93 (0.42)	49.8 (28.65)	41 (44)	2 (40)	
**Marital status**							**<0.001** ^b^
Single	226 (20)	0.84 (0.23)	0.91 (0.21)	65.75 (23.01)	70 (32.5)	101 (44.69)	
Married	572 (50.62)	0.77 (0.3)	0.89 (0.35)	60.74 (21.44)	62 (28)	192 (33.57)	
Divorced/separated	75 (6.64)	0.78 (0.27)	0.67 (0.47)	60.13 (21.65)	54 (30)	18 (24)	
Widowed	255 (22.57)	0.59 (0.34)	0.86 (0.21)	55.16 (21.11)	63 (26)	31 (12.16)	
**Smoking status**							**<0.001** ^a^
Yes	282 (24.96)	0.83 (0.25)	0.91 (0.23)	64.97 (20.95)	70 (29)	108 (38.3)	
No	847 (74.96)	0.71 (0.32)	0.82 (0.43)	58.93 (22.07)	60 (31)	235 (27.74)	
**Net monthly income household**							**<0.001** ^b^
Less than 550 €	106 (9.38)	0.69 (0.31)	0.77 (0.36)	57.82 (20.66)	55.5 (26.3)	19 (17.92)	
551–1,300 €	523 (46.28)	0.71 (0.31)	0.82 (0.42)	58.38 (21.85)	60 (31)	130 (24.86)	
1,301–2,250 €	186 (16.46)	0.78 (0.31)	0.89 (0.3)	61.9 (22.93)	65 (31)	70 (37.63)	
2,251–3,450 €	72 (6.37)	0.81 (0.31)	0.93 (0.26)	66.01 (22.93)	72.5 (33.3)	35 (48.61)	
3,451 + €	19 (1.68)	0.92 (0.14)	1 (0.11)	71.63 (14.37)	70 (21)	12 (63.16)	
**Educational level**							**<0.001** ^b^
Low	552 (48.85)	0.67 (0.33)	0.76 (0.38)	55.59 (21.61)	56 (30)	114 (20.65)	
Medium	463 (40.97)	0.79 (0.28)	0.89 (0.29)	63.45 (21.71)	66 (31)	165 (35.64)	
High	115 (10.18)	0.88 (0.21)	1 (0.14)	70.87 (18.43)	74 (21)	64 (55.65)	

a, Mann-Whitney U.

b, Kruskal Wallis H.

Educational level: According to the International Standard Classification of Education (ISCED); Low educational level (Early childhood education and Primary education), Medium educational level (Lower secondary education, Upper secondary education and Post-secondary non-tertiary education) and High educational level (tertiary education).

A total of 1130 COPD patients participated in the survey. Of these, 550 (48.67%) were males and 580 (51.33%) were females. Mean (SD) EQ-5D-5L utility for the whole sample was 0.74 (0.30). In general terms, men reported higher scores in this utility [0.79 (0.27)] than women [0.69 (0.32)]. The VAS score was slightly higher in men compared with women, 61.86 (21.65) and 59.16 (22.12), respectively.

Age had a relevant effect in the utility index and VAS score. In this regard, older age groups reported much lower scores on both measures than younger groups. Results varied by region; higher scores in the utility were observed in La Rioja and the autonomous city of Ceuta, 0.88 (0.23) and 0.85 (0.16) respectively. On the other hand, worst results were observed in Cantabria and Castile-La Mancha, where the utility of the EQ-5D-5L was 0.63 (0.43) and 0.65 (0.39) respectively.

Twenty-five percent (25%) of the sample were regular smokers. This group reported higher scores in the utility index of EQ-5D-5L and the VAS score compared with the non-smoker group. As expected, the two HRQoL measures were higher as the monthly incomes and educational level were higher. Besides, means of the EQ-5D-5L utilities showed significant differences (*P*<0.01) among the different sub-groups of demographic variables, except with the region variable (0.09). Results by sex are shown in Table 2.

**Table 2:** Study sample characteristics, EQ-5D-5L male and female population norms

	**n = 1130**		**EQ-5D-5L Index**				**EQ-5D-5L VAS**				**Ceiling Effect**	
	**Male**	**Female**	**Male**		**Female**		**Male**		**Female**		**Male**	**Female**
	**n**	**n**	**Mean (SD)**	**Median (IQR)**	**Mean (SD)**	**Median (IQR)**	**Mean (SD)**	**Median (IQR)**	**Mean (SD)**	**Median (IQR)**	**%**	**%**
**Age group**												
15–17	9	6	1 (0)	1 (0)	0.97 (0.04)	1 (0.08)	84.67 (15.48)	82 (20)	91.83 (4.62)	91.5 (6.5)	100.00	66.67
18–29	20	23	0.97 (0.08)	1 (0)	0.97 (0.08)	1 (0)	85.5 (9.56)	84.5 (13.5)	81.83 (19.07)	87 (15)	85.00	78.26
30–39	28	43	0.96 (0.11)	1 (0.07)	0.89 (0.13)	0.93 (0.17)	76.25 (14.43)	76 (18.75)	66.21 (22.42)	71 (35)	75.00	41.86
40–49	45	59	0.87 (0.2)	0.91 (0.18)	0.84 (0.26)	0.93 (0.23)	60.21 (22.23)	61 (25)	69.54 (18.6)	74 (23)	42.22	45.76
50–59	76	82	0.84 (0.2)	0.91 (0.23)	0.73 (0.31)	0.83 (0.34)	59.05 (22.6)	60 (39.75)	60.99 (21.19)	60.5 (27.5)	31.58	23.17
60–69	129	116	0.85 (0.21)	0.91 (0.22)	0.73 (0.27)	0.82 (0.37)	63.9 (18.66)	68.5 (28)	58.97 (19.99)	57 (25)	39.53	21.55
70–79	123	142	0.77 (0.3)	0.89 (0.34)	0.64 (0.32)	0.7 (0.37)	59.97 (19.62)	62 (25.75)	52.99 (20.68)	55 (29.25)	34.15	12.68
80–89	106	96	0.64 (0.37)	0.76 (0.42)	0.47 (0.38)	0.57 (0.55)	54.93 (23.35)	59 (37)	51.43 (21.75)	50 (32.5)	19.81	8.33
90 +	14	13	0.58 (0.3)	0.63 (0.53)	0.31 (0.32)	0.29 (0.46)	51.31 (27.27)	50 (38)	43.33 (21.19)	42.5 (28.5)	14.29	0.00
**Region**												
Andalusia	60	63	0.77 (0.29)	0.87 (0.33)	0.6 (0.39)	0.72 (0.59)	58.45 (21.97)	61 (36)	53.56 (21.44)	50 (35)	26.67	14.29
Aragon	19	23	0.77 (0.31)	0.89 (0.27)	0.71 (0.33)	0.84 (0.4)	64.89 (19.55)	65 (51)	54.61 (15.27)	35 (15)	42.11	26.09
Principality of Asturias	28	36	0.73 (0.35)	0.83 (0.35)	0.68 (0.32)	0.76 (0.4)	61.93 (21.42)	62.5 (29)	57 (18.57)	60 (20.75)	35.71	25.00
Balearic Islands	20	11	0.81 (0.24)	0.97 (0.4)	0.73 (0.28)	0.89 (0.5)	65.2 (26.84)	75.5 (45.75)	65.64 (25.09)	71 (40)	50.00	18.18
Canarias	27	47	0.72 (0.35)	0.85 (0.4)	0.67 (0.28)	0.75 (0.35)	58.11 (21.28)	61 (22)	59.98 (21.24)	57 (29)	29.63	12.77
Cantabria	9	20	0.81 (0.29)	0.91 (0.26)	0.55 (0.47)	0.69 (0.94)	52.56 (16.85)	50 (22)	48.7 (20.24)	48 (25.75)	33.33	30.00
Castile and Leon	39	34	0.85 (0.16)	0.89 (0.3)	0.79 (0.2)	0.82 (0.25)	55.97 (20.37)	52 (35)	62.26 (21.76)	60.5 (38)	35.90	23.53
Castile-La Mancha	31	38	0.8 (0.34)	0.91 (0.15)	0.54 (0.41)	0.56 (0.63)	62.26 (25.03)	65 (44)	53.45 (24.19)	56 (39.75)	48.39	23.68
Catalonia	77	72	0.79 (0.25)	0.86 (0.32)	0.74 (0.27)	0.83 (0.42)	65.88 (21.16)	70 (29)	62.49 (22.6)	62 (32)	28.57	27.78
Community of Valencia	42	41	0.79 (0.36)	0.97 (0.23)	0.68 (0.3)	0.74 (0.3)	63.19 (20.05)	67.5 (25.75)	58.59 (20.01)	60 (21.5)	50.00	17.07
Extremadura	35	21	0.84 (0.17)	0.89 (0.24)	0.78 (0.248)	0.84 (0.36)	56.62 (21.37)	60.5 (25.75)	53.43 (27.25)	51 (41.5)	34.29	28.57
Galicia	33	39	0.73 (0.37)	0.91 (0.4)	0.72 (0.284)	0.8 (0.47)	57.18 (19.66)	61 (26.5)	64.54 (20.1)	66 (25)	30.30	25.64
Community of Madrid	37	41	0.81 (0.23)	0.89 (0.29)	0.71 (0.34)	0.84 (0.43)	66.11 (20.98)	69 (33.5)	63.05 (26.11)	69 (37)	37.84	24.39
Murcia Region	27	24	0.85 (0.21)	0.97 (0.24)	0.77 (0.22)	0.81 (0.43)	64.59 (22.65)	70 (29)	60.58 (23.12)	54.5 (31.5)	44.44	29.17
Community of Navarre	25	22	0.81 (0.27)	0.91 (0.29)	0.8 (0.186)	0.85 (0.2)	60.6 (19.78)	66 (28.5)	62.55 (16.16)	69 (22)	36.00	13.64
Basque Country	22	34	0.82 (0.26)	0.9 (0.28)	0.66 (0.45)	0.86 (0.62)	66.18 (21.17)	64.5 (33.75)	64.47 (23.87)	70 (31)	45.45	41.18
La Rioja	10	9	0.97 (0.74)	1 (0.04)	0.78 (0.318)	0.92 (0.45)	83.8 (11.13)	85 (14.25)	69.78 (22.15)	82 (44.5)	80.00	44.44
Ceuta	6	3	0.91 (0.14)	0.96 (0.16)	0.73 (0.186)	0.74 (0.37)	66.17 (28.99)	77.5 (33.5)	48 (31.58)	61 (59)	50.00	0.00
Melilla	3	2	0.84 (0.22)	0.93 (−)	0.79 (0.3)	0.79 (−)	56.67 (37.07)	41 (−)	39.5 (13.44)	39.5 (−)	33.33	50.00
**Marital status**												
Single	122	104	0.87 (0.2)	0.91 (0.18)	0.81 (0.27)	0.91 (0.27)	65.5 (23.59)	73 (31)	66.03 (22.45)	69 (35)	48.36	40.38
Married	335	237	0.78 (0.3)	0.91 (0.32)	0.74 (0.3)	0.84 (0.41)	60.8 (21.21)	64 (27)	60.66 (21.79)	61 (30)	37.91	27.43
Divorced/separated	60	195	0.71 (0.28)	0.88 (0.23)	0.55 (0.35)	0.83 (0.26)	59.76 (20.33)	68.5 (28.75)	53.75 (21.2)	60 (30)	16.67	10.77
Widowed	32	43	0.82 (0.24)	0.78 (0.35)	0.75 (0.29)	0.64 (0.53)	63.13 (20.75)	64.5 (27.75)	57.91 (22.27)	51 (29)	31.25	18.60
**Smoking status**												
Yes	157	125	0.85 (0.21)	0.91 (0.2)	0.79 (0.28)	0.89 (0.28)	64.59 (19.71)	70 (27)	65.45 (22.49)	70 (30)	42.04	33.60
No	393	454	0.77 (0.3)	0.89 (0.33)	0.66 (0.33)	0.76 (0.4)	60.74 (22.34)	63 (30.75)	57.4 (21.74)	58 (29)	35.62	20.93
**Net Monthly income household**												
Less than 550 €	39	67	0.87 (0.14)	0.91 (0.23)	0.59 (0.34)	0.67 (0.34)	67.08 (20.48)	71 (30)	52.43 (18.9)	51 (19)	35.90	7.46
551–1,300 €	250	273	0.76 (0.29)	0.86 (0.35)	0.67 (0.32)	0.76 (0.38)	57.85 (22.22)	61.5 (33.5)	58.84 (21.54)	59 (30)	29.60	20.51
1,301–2,250 €	100	86	0.84 (0.25)	0.91 (0.22)	0.72 (0.37)	0.89 (0.35)	64.78 (21.09)	70 (27)	58.51 (24.62)	60 (40)	45.00	29.07
2,251–3,450 €	39	33	0.83 (0.31)	1 (0.18)	0.79 (0.31)	0.91 (0.28)	68.87 (19.83)	72 (25)	62.64 (26.02)	73 (43)	53.85	42.42
3,451 + €	9	10	0.89 (0.19)	1 (0.25)	0.95 (0.07)	1 (0.1)	68.56 (15.99)	69 (24)	74.4 (12.96)	78.5 (17.75)	66.67	60.00
**Educational level**												
Low	272	280	0.75 (0.31)	0.86 (0.35)	0.59 (0.33)	0.68 (0.44)	58.13 (21.6)	60 (31.75)	53.18 (21.37)	53 (29)	29.41	12.14
Medium	222	241	0.84 (0.24)	0.91 (0.23)	0.75 (0.31)	0.86 (0.35)	64.3 (21.85)	69 (31)	62.68 (21.59)	62 (31)	41.89	29.88
High	56	59	0.87 (0.24)	1 (0.18)	0.9 (0.19)	1 (0.14)	69.46 (17.8)	72.5 (19)	72.22 (19.07)	77 (26)	58.93	52.54

Educational level: According to the International Standard Classification of Education (ISCED); Low educational level (Early childhood education and Primary education), Medium educational level (Lower secondary education, Upper secondary education and Post-secondary non-tertiary education) and High educational level (tertiary education).

The score in the utility index of EQ-5D-5L reported by males was higher than the reported by females in the 9 age groups and in all the regions. These differences were detected regardless marital and smoking status. However, this tendency was not observed in the group with higher monthly incomes and higher educational level, where women reported better HRQoL. In the VAS score, the results did not entirely follow the tendency of the utility: men reported higher scores in 6 of the 9 age groups, and in 14 of the 19 regions. When educational level was low or medium, men reported higher VAS scores than women, but women reported better health status than men when educational level was high.

Table 3 shows the distribution of EQ-5D-5L dimensions by gender and age groups. The frequency of the level of problem 5 was always higher in the female group.

**Table 3:** Percentage frequency distributions of EQ-5D-5L dimensions by gender and age group

Level	**Mobility**			**Self-care**			**Usual activities**			**Pain/discomfort**			**Anxiety/depression**		
	**Total**	**Male**	**Female**	**Total**	**Male**	**Female**	**Total**	**Male**	**Female**	**Total**	**Male**	**Female**	**Total**	**Male**	**Female**
All															
1	54.6	60.0	49.5	77.8	81.5	74.3	62.5	68.8	56.6	42.9	52.7	33.6	65.1	73.3	57.2
2	17.1	16.5	17.6	8.9	9.1	8.8	15.2	12.9	17.4	22.8	22.8	22.7	15.7	13.4	17.9
3	14.6	12.4	16.7	6.5	4.4	8.4	11.3	8.9	13.6	21.1	16.1	25.8	11.9	8.3	15.3
4	11.0	8.9	12.9	3.2	1.6	4.7	5.5	4.4	6.6	11.7	6.9	16.2	5.2	2.7	7.6
5	2.7	2.2	3.3	3.6	3.5	3.8	5.3	4.7	5.9	1.1	0.7	1.4	1.1	0.9	1.4
15–17	**Total**	Male	Female	**Total**	Male	Female	**Total**	Male	Female	**Total**	Male	Female	**Total**	Male	Female
1	100.0	100.0	100.0	100.0	100.0	100.0	100.0	100.0	100.0	100.0	100.0	100.0	86.7	100.0	66.7
2	0.0	0.0	0.0	0.0	0.0	0.0	0.0	0.0	0.0	0.0	0.0	0.0	6.7	0.0	16.7
3	0.0	0.0	0.0	0.0	0.0	0.0	0.0	0.0	0.0	0.0	0.0	0.0	6.7	0.0	16.7
4	0.0	0.0	0.0	0.0	0.0	0.0	0.0	0.0	0.0	0.0	0.0	0.0	0.0	0.0	0.0
5	0.0	0.0	0.0	0.0	0.0	0.0	0.0	0.0	0.0	0.0	0.0	0.0	0.0	0.0	0.0
18–29	**Total**	Male	Female	**Total**	Male	Female	**Total**	Male	Female	**Total**	Male	Female	**Total**	Male	Female
1	100.0	100.0	100.0	100.0	100.0	100.0	97.7	95.0	100.0	83.7	85.0	82.6	90.7	95.0	87.0
2	0.0	0.0	0.0	0.0	0.0	0.0	2.3	5.0	0.0	11.6	15.0	8.7	4.7	0.0	8.7
3	0.0	0.0	0.0	0.0	0.0	0.0	0.0	0.0	0.0	2.3	0.0	4.3	2.3	0.0	4.3
4	0.0	0.0	0.0	0.0	0.0	0.0	0.0	0.0	0.0	2.3	0.0	4.3	2.3	5.0	0.0
5	0.0	0.0	0.0	0.0	0.0	0.0	0.0	0.0	0.0	0.0	0.0	0.0	0.0	0.0	0.0
30–39	**Total**	Male	Female	**Total**	Male	Female	**Total**	Male	Female	**Total**	Male	Female	**Total**	Male	Female
1	94.4	96.4	93.0	98.6	96.4	100.0	87.3	92.9	83.7	64.8	82.1	53.5	76.1	89.3	67.4
2	0.0	0.0	0.0	1.4	3.6	0.0	8.5	0.0	14.0	15.5	10.7	18.6	14.1	7.1	18.6
3	5.6	3.6	7.0	0.0	0.0	0.0	4.2	7.1	2.3	16.9	7.1	23.3	4.2	0.0	7.0
4	0.0	0.0	0.0	0.0	0.0	0.0	0.0	0.0	0.0	2.8	0.0	4.7	5.6	3.6	7.0
5	0.0	0.0	0.0	0.0	0.0	0.0	0.0	0.0	0.0	0.0	0.0	0.0	0.0	0.0	0.0
40–49	**Total**	Male	Female	**Total**	Male	Female	**Total**	Male	Female	**Total**	Male	Female	**Total**	Male	Female
1	78.8	80.0	78.0	93.3	95.6	91.5	81.7	88.9	76.3	59.6	64.4	55.9	63.5	60.0	66.1
2	11.5	13.3	10.2	3.8	2.2	5.1	9.6	6.7	11.9	26.9	28.9	25.4	19.2	20.0	18.6
3	3.8	2.2	5.1	1.0	2.2	0.0	2.9	0.0	5.1	8.7	4.4	11.9	9.6	11.1	8.5
4	4.8	4.4	5.1	1.9	0.0	3.4	3.8	2.2	5.1	2.9	2.2	3.4	4.8	4.4	5.1
5	1.0	0.0	1.7	0.0	0.0	0.0	1.9	2.2	1.7	1.9	0.0	3.4	2.9	4.4	1.7
50–59	**Total**	Male	Female	**Total**	Male	Female	**Total**	Male	Female	**Total**	Male	Female	**Total**	Male	Female
1	62.7	68.4	57.3	86.7	88.2	85.4	67.3	70.1	64.6	44.3	56.6	32.9	60.8	64.5	57.3
2	15.2	15.8	14.6	4.4	5.3	3.7	13.2	10.4	15.9	20.3	21.1	19.5	17.1	21.1	13.4
3	13.3	10.5	15.9	5.7	3.9	7.3	12.6	13.0	12.2	24.1	15.8	31.7	14.6	14.5	14.6
4	7.6	5.3	9.8	0.6	1.3	0.0	2.5	1.3	3.7	10.8	6.6	14.6	6.3	0.0	12.2
5	1.3	0.0	2.4	2.5	1.3	3.7	3.1	2.6	3.7	0.6	0.0	1.2	1.3	0.0	2.4
60–69	**Total**	Male	Female	**Total**	Male	Female	**Total**	Male	Female	**Total**	Male	Female	**Total**	Male	Female
1	58.4	66.7	49.1	86.5	90.7	81.9	71.0	75.2	66.4	44.1	60.0	37.9	63.3	77.5	47.4
2	18.8	13.2	25.0	6.9	4.7	9.5	13.1	11.6	14.7	22.0	25.2	24.3	17.1	13.2	21.6
3	15.5	14.7	16.4	4.5	3.1	6.0	10.6	8.5	12.9	20.4	10.4	25.2	13.1	7.8	19.0
4	6.9	5.4	8.6	1.6	0.8	2.6	4.5	3.9	5.2	12.7	4.3	11.7	4.9	1.6	8.6
5	0.4	0.0	0.9	0.4	0.8	0.0	0.8	0.8	0.9	0.8	0.0	1.0	1.6	0.0	3.4
70–79	**Total**	Male	Female	**Total**	Male	Female	**Total**	Male	Female	**Total**	Male	Female	**Total**	Male	Female
1	42.6	52.0	34.5	72.8	79.7	66.9	53.6	66.7	42.3	33.5	48.4	20.4	65.0	72.6	58.5
2	25.7	25.2	26.1	14.3	13.8	14.8	21.9	18.7	24.6	27.8	25.0	30.3	14.3	12.1	16.2
3	15.8	11.4	19.7	6.4	2.4	9.9	14.7	7.3	21.1	22.6	16.1	28.2	13.9	7.3	19.7
4	11.7	7.3	15.5	3.0	0.0	5.6	4.2	2.4	5.6	14.3	8.1	19.7	5.6	5.6	5.6
5	4.2	4.1	4.2	3.4	4.1	2.8	5.7	4.9	6.3	1.1	0.8	1.4	0.4	0.8	0.0
80–89	**Total**	Male	Female	**Total**	Male	Female	**Total**	Male	Female	**Total**	Male	Female	**Total**	Male	Female
1	25.7	31.1	19.8	52.5	58.5	45.8	37.6	46.2	28.1	25.7	34.0	16.7	61.8	71.3	51.0
2	20.3	22.6	17.7	15.3	17.9	12.5	21.3	18.9	24.0	23.3	25.5	20.8	17.6	13.9	21.9
3	22.3	17.9	27.1	14.4	9.4	19.8	15.3	13.2	17.7	30.2	27.4	33.3	11.8	8.3	15.6
4	25.2	22.6	28.1	7.4	4.7	10.4	10.9	7.5	14.6	18.8	10.4	28.1	5.4	0.9	10.4
5	6.4	5.7	7.3	10.4	9.4	11.5	14.9	14.2	15.6	2.0	2.8	1.0	1.5	1.9	1.0
90 or more	**Total**	Male	Female	**Total**	Male	Female	**Total**	Male	Female	**Total**	Male	Female	**Total**	Male	Female
1	11.1	21.4	0.0	22.2	35.7	7.7	14.8	21.4	7.7	27.6	33.3	21.4	56.7	66.7	46.7
2	7.4	7.1	7.7	11.1	14.3	7.7	3.7	7.1	0.0	24.1	26.7	21.4	6.7	0.0	13.3
3	40.7	42.9	38.5	22.2	21.4	23.1	22.2	21.4	23.1	27.6	26.7	28.6	13.3	13.3	13.3
4	29.6	21.4	38.5	22.2	14.3	30.8	37.0	42.9	30.8	6.9	0.0	14.3	3.3	6.7	0.0
5	11.1	7.1	15.4	22.2	14.3	30.8	22.2	7.1	38.5	0.0	0.0	0.0	0.0	0.0	0.0

**Fig. 1:**
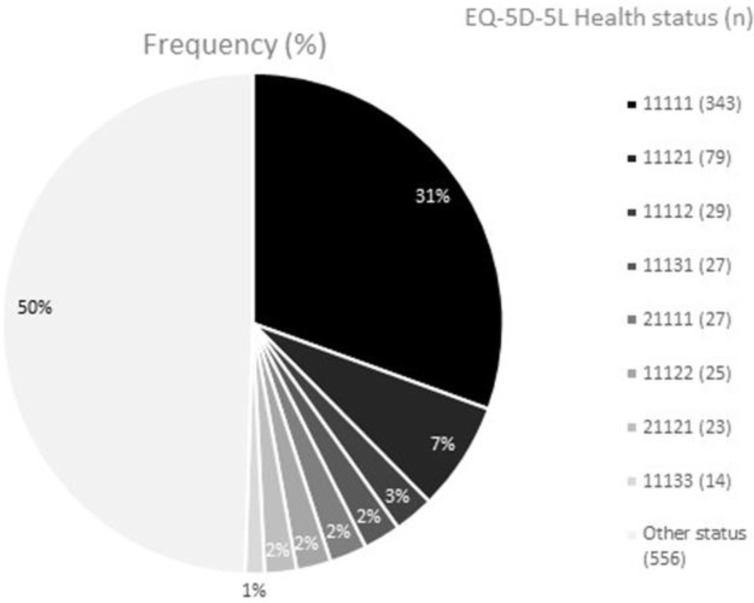
Spanish distribution of EQ-5D-5L Health Status (n=1130)

Distribution of the health status in Spanish COPD patients can be observed in Fig. 1. The most frequent health status was 11111. More than 30% of the sample reported this health status. The second and third most frequent health states were 11121 and 11112 respectively.

Ceiling effect can be observed in Table 1 and 2, and Fig. 1. Of 1130 participants, 343 reported perfect health status, which means 30.35% of the total sample. Ceiling effect was higher among males (37.45%) than among females (23.62%). It was reduced as the age was increased, and was increased, as the monthly incomes and educational level were higher.

## Discussion

To our knowledge, this is the first article that aims to provide normative values of EQ-5D-5L for Spanish people affected by COPD. Spanish men affected by COPD reported better health status than women. These results are consistent with previous studies that reported worse HRQoL in women with COPD compared with men (10, 11). This gender difference was higher in the EQ-5D-5L utility index (14%) and lower in the VAS score (4%). Results in previous studies also showed the same discrepancy between males and females in other diseases, such as cancer (12) or diabetes (13). Women and men might understand or interpret differently their own health status and there could be another important variable not assessed in EQ-5D-5L that could strongly influence their self-reported health status.

Gender differences were reduced, as the net monthly incomes and educational level were higher. In this regard, bigger sex differences were observed in those patients with less than 550€ per month and in those with low educational level. These results support the notion of an association between knowledge about the own disease and the ability to handle the disease better (14) and are consistent with previous studies that reported a positive association between educational level and knowledge about the own disease (15, 16). Therefore, the current study supports the relevance of health education as a tool for the management of disease.

One of the most unexpected findings of the current study was that smokers reported higher scores in the utility index and the VAS compared with non-smokers. However, one limitation of the current study is that there was no differentiation between patients that never smoked and those that quit smoking. In this regard, the observed results could be due to a high percent of ex-smokers in the non-smokers group.

In the current study, 343 participants (30.35% of the COPD sample) reported perfect health status. This result is higher respect to other studies. A multi-country study reported a ceiling effect of only 7% in the EQ-5D-5L and 8.5% in the EQ-5D-3L (9). However, those ceiling effects are much lower than the observed in the EQ-5D-3L for Spanish people with COPD (17), which was 22% (moderate COPD 29.6%, severe COPD 20%, and very severe COPD 10.6%). According to dimensions, the greatest ceiling effect (77.8%) was observed in the dimension “self-care”, whereas the lowest was found in the dimension “pain/discomfort” (42.9%).

Studies providing normative values of HRQoL in special populations contribute allowing comparisons between specific pathologic or notpathologic populations and general population, helping the development and planning of health policy (18, 19). Normative values allow researchers to estimate the clinical relevance of a treatment, training or intervention (20, 21) and may be a useful tool in interpreting patient-reported outcome results (22).

The current study has several limitations. The most relevant limitation is the lack of another measure that could classify patients according to the severity of the disease. The second limitation is the lack of an algorithm specifically designed for EQ-5D-5L in Spanish populations, so the Spanish utility index of the 5 level version of EQ-5D is the result of a “crosswalk” from the previous 3 level version. In spite of these 2 limitations, this study meets the main mentioned objective, which is the setting of normative values for the Spanish population affected by COPD.

## Conclusion

The current study provides normative values of EQ-5D-5L for Spanish patients suffering from COPD. Mean (SD) EQ-5D-5L utility and VAS score were 0.74 (0.30) and 60.46 (21.93) respectively. Men reported better health status than women. As educational level and monthly incomes were higher, gender differences were lower and HRQoL was better.

## Ethical considerations

Ethical issues (Including plagiarism, informed consent, misconduct, data fabrication and/or falsification, double publication and/or submission, redundancy, etc.) have been completely observed by the authors.
